# Burnout among public health physicians and residents in Canada following the COVID-19 pandemic: A cross-sectional study

**DOI:** 10.1371/journal.pmen.0000527

**Published:** 2025-12-23

**Authors:** Syeda Aiman Fatima, Xi Yu Hao, Diana Sun, Itai Malkin, Elizabeth Alvarez, Laura N. Anderson, David Edward-Ooi Poon, Japteg Singh, Emma Apatu, Chris P. Verschoor, Thomas Piggott, Emily Belita, Sheila A. Boamah, Arielle Sutton, Zayya Zendo, Jessica P. Hopkins

**Affiliations:** 1 Dalla Lana School of Public Health, University of Toronto, Toronto, Canada; 2 Public Health and Preventive Medicine Residency Program, Northern Ontario School of Medicine, Sudbury, Canada; 3 Public Health and Preventive Medicine Residency Program, University of Toronto, Toronto, Canada; 4 Department of Health Research Methods, Evidence, and Impact, McMaster University, Hamilton, Canada; 5 School of Public Health, University of Saskatchewan, Saskatoon, Canada; 6 McGill University, Montreal, Canada; 7 Health Sciences North Research Institute, Sudbury, Canada; 8 Department of Family Medicine, Queen’s University, Kingston, Canada; 9 School of Nursing, McMaster University, Hamilton, Canada; 10 MD Program, Faculty of Medicine and Dentistry, Schulich School of Medicine, Western University, London, Canada; 11 Public Health Ontario, Toronto, Canada; Uganda Martyrs University, UGANDA

## Abstract

Previous research identified high levels of burnout in the Canadian public health workforce during the COVID-19 pandemic. This study presents the prevalence of burnout, associated participant demographic and workplace characteristics, and associated secondary outcomes among Canadian public health physicians and residents one year after the end of the COVID-19 pandemic. Data were collected using an online survey distributed through Canadian public health associations and professional networks between April and May 2024. Validated tools were used to measure burnout (Oldenburg Burnout Inventory (OLBI)), screen for anxiety (GAD-2) and depression (PHQ-2), and professional fulfillment (Stanford Professional Fulfillment Index). Additional binary (yes/no) questions were asked on workplace safety topics (e.g., threats, assaults, being bullied) and professional plans. Fisher’s exact test and logistic regressions were used to model the association between burnout and sequelae of burnout, including symptoms of depression and anxiety, and professional fulfillment. Among 118 physicians who completed the OLBI, the prevalence of burnout was 63.6%. Additionally, 41.2% of physicians reported being threatened, assaulted or bullied during the pandemic. Physicians who screened positive for anxiety (19.3%) and depression (7.6%) had higher odds of burnout (OR 4.79, 95% CI 1.29-26.90, p = 0.01 and OR 2.10, 95% CI 0.38-21.65, p = 0.48, respectively). Moreover, physicians who had low levels of professional fulfillment (84.9%) also had higher odds of burnout (OR 12.5, 95% CI 3.21-72.76, p < 0.001). The prevalence of burnout among Canadian public health physicians and residents remains high post-pandemic and was associated with symptoms of depression, anxiety and low professional fulfillment. By implementing interventions to prevent and mitigate burnout, and promote recovery, the public health system will be better positioned to recruit and retain physicians to serve the population.

## Introduction

Burnout is an occupational health syndrome characterized by emotional exhaustion, depersonalization, and diminished sense of achievement from the chronic exposure to stressors in the workplace [1]. It has been associated with poor mental health outcomes including anxiety, depression, and suicidal ideation [2,3]. Furthermore, it can also have organizational consequences such as absenteeism, job dissatisfaction, interpersonal strain, and reduced job performance [4].

The COVID-19 pandemic has worsened burnout among many health professionals. Since the start of the pandemic in 2020, several studies have described the prevalence of burnout among Canadian healthcare providers [5–7]. Sixty-five percent of physicians practicing in a cardiovascular centre at two quaternary referral hospitals in Toronto, Canada were found to have burnout between November 2018 and January 2019 [6]. In a cross-sectional survey in Ontario, Canada in 2020 and repeated in 2021, 66% and 73% of physicians, respectively, experienced at least some level of burnout [5]. In 2021, 53% of physicians participating in the National Physician Health Survey in Canada reported feelings of burnout, a 22% increase in four years [8].

Given the magnitude and intensity of the public health response to the COVID-19 crisis, public health physicians and other public health workers experienced unusually high work demands for the duration of the pandemic (2020–2023). However, few studies have measured burnout among the Canadian public health workforce [9].

In January 2023, we conducted a national survey of 2,079 Canadian public health workers and found that 79% met the criteria for burnout [10]. In addition, meeting the criteria for burnout was associated with a greater intention to leave public health or retire early [10]. We found that burnout in the public health workforce was associated with five or more years of work experience, redeployment to the pandemic response, being harassed during the pandemic, feeling unsafe in the workplace, and not being offered workplace supports [10]. This is consistent with findings from Belita *et al*. who conducted a Canada-wide survey in the same time period and found that those who felt bullied, threatened, or harassed because of work had a higher intention to leave [9].

To date, studies on and strategies to address burnout in physicians in Canada have largely focused on physicians who provide direct clinical care [5,11]. Findings from these studies may not be applicable to the Canadian public health physician population who provide organizational and medical leadership to multi-disciplinary organizations to improve the health of communities. Our previous cross-sectional study and the study by Belita *et al.* examined burnout among the wider public health workforce, but there are no other sources of data examining burnout specifically among public health physicians and residents in Canada [9,10]. This study builds on our previous work to examine the prevalence of burnout by assessing any change in the prevalence of burnout since our first study, determining associations between participant demographic and workplace characteristics and burnout, and exploring sequelae of burnout including depression, anxiety and professional fulfillment among public health physician leaders and public health and preventive medicine residents in Canada.

## Methods

### Study design

A cross-sectional online survey was conducted (see S1 Appendix for the CHERRIES reporting checklist). We invited Canadian public health physician leaders working in a local or regional health authority (i.e., Medical Health Officers, Associate Medical Health Officers, and physician CEOs), public health and preventive medicine residency program directors, and public health and preventive medicine residents to participate in an online survey from April 8 to May 31, 2024. The survey was available in English and French through Surveys@PHO which is a password protected web-based application at Public Health Ontario. Invitation emails were distributed through Canadian local/regional, provincial and national public health associations and professional groups, and to program directors and lead/senior/representative residents of every public health and preventive medicine residency program in Canada. A convenience sample using broad outreach across national, provincial/territorial and local professional associations and networks was used as there was no registry or census of public health physicians in Canada. Residents were additionally recruited through snowball sampling. Participation in the study was voluntary, and survey responses were anonymized and reported in aggregate. The following physicians were eligible to participate in the survey: current Medical Officers of Health/Medical Health Officers, Associate Medical Officers of Health/Associate Medical Health Officers, Physician Leaders and CEOs of a public health unit/regional health authority, Public Health and Preventive Medicine Residency Program Directors/Assistant Program Directors, and Public Health and Preventive Medicine Residents in Canada. Individuals not currently practicing public health in Canada were not eligible to participate and were excluded from the study as no systematic way to identify and recruit them exists.

To measure the prevalence of burnout, a minimum sample size was calculated *a priori* using the following equation:


$$N\;=\;Z^{\text{2}}P(\text{1}-P)/d^{\text{2}}\;$$

where confidence level (Z) of 95% margin of error (d) of 5% and estimated prevalence (P) of burnout of 79% (based on our previous study, assuming a population size of approximately 200 physicians [10]).

Therefore, a minimum sample of at least 113 physicians was determined.

### Study measures

The survey collected information on the following variables: demographics, work characteristics (e.g., feelings of safety in the workplace, intention to leave/retire), burnout, symptoms of depression and anxiety, professional fulfillment, and self-valuation and compassion through closed- and character-limited open-ended questions (see S2 Appendix for the full survey). The following key factors that could increase risk or mitigate burnout were explored: age, gender, years of work experience, being a caregiver to children less than 18 years or adult dependents, identifying as racialized or a person of colour, symptoms of depression and anxiety, and professional fulfillment.

The primary outcome of burnout was measured using the validated Oldenburg Burnout Inventory (OLBI), a 16-item Likert-type questionnaire consisting of two subscales: exhaustion and disengagement [12]. Exhaustion refers to general feelings of emptiness, work overload, strong need for rest, and cognitive, emotional and physical fatigue [1]. Disengagement relates to feelings of withdrawal, and negative attitudes and behaviours towards work [1]. Each burnout subscale consisted of eight items: four positively worded and four negatively worded statements presented in mixed order with responses constructed on a 4-point Likert-scale ranging from totally disagree to totally agree. Scoring for negatively worded items was reversed, and the scores were calculated by adding the average of the items in each subscale as recommended. Exhaustion was defined as a score of 2.25 or higher on the exhaustion subscale, while disengagement was defined as a score of 2.10 or higher on the disengagement subscale [1]. Overall burnout was a binary measure, and it was defined as physicians meeting cut-off scores for both exhaustion and disengagement, suggestive of “high burnout” [1].

Several potential sequelae of burnout were also measured. To assess for symptoms of anxiety and depression, brief screening tools were included in the survey. The Patient Health Questionnaire-2 (PHQ-2) consists of the first two items of the PHQ-9, which are considered the two core criteria for depressive disorders [13]. The Generalized Anxiety Disorder 2-item (GAD-2) consists of the first two items of the GAD-7, which are considered core criteria for diagnosing an anxiety disorder [13]. Each item for both screening tools was rated on a 4-point Likert scale (0–3), with increasing scores indicating greater symptom severity [13]. Total scores range from 0 to 6, with a total score greater than or equal to 3 suggestive, but not diagnostic, of depression or generalized anxiety disorder [13]. Using a cut-off of 3 or greater, the PHQ-2 has a sensitivity of 0.72 (95% CI 0.67-0.77) and specificity of 0.85 (95% CI 0.83-0.87) [14]. This compares to PHQ-9 scores greater than 10 having both sensitivity and specificity of 0.88 for major depressive disorder [15]. Using a cut-off of 3 or greater, the GAD-2 has a sensitivity of 0.76 (95% CI 0.55-0.89) and specificity of 0.81 (95% CI 0.60-0.92) [16]. This compares to GAD-7 scores of 8 or greater that have a sensitivity of 0.83 (95% CI 0.71-0.91) and specificity 0.84 (95% CI 0.70-0.92) for generalized anxiety disorder [16]. The two-item scales were selected based on feedback in the development and testing phase of the survey that suggested the PHQ-9 and GAD-7 were too long and may not be acceptable to users due to the more detailed data collection on mental health.

The Stanford Professional Fulfillment Index (SPFI) is a 6-item instrument to assess physicians’ professional fulfillment, while the self-valuation scale is a 4-item instrument that assesses deferment of self-care to meet work demands and harsh response to personal imperfections and errors [17,18]. Both instruments were rated on a 5-point (0–4) Likert scale (summative score range, 0–16). Based on the literature, the cut-off score for determining presence of moderate to high professional fulfillment using the SPFI scale is an average-item score of 3.0 or greater [17]. For determining levels of self-valuation, scores of 8 or less were categorized as low levels of self-valuation and scores of 9 or more as moderate or high [18].

### Analysis

Quantitative data were analyzed using R, version 4.4.1. Descriptive statistical analyses were conducted to provide information on participant characteristics, including the prevalence of the three OLBI measures (burnout, exhaustion and disengagement), subgroup analyses of burnout by age, gender and independently practicing physicians vs. resident trainees were conducted as well as prevalence of secondary outcomes like professional fulfillment, self-valuation, and screening criteria for depression and anxiety. Fisher’s exact test was used to detect any significant association between burnout and secondary outcomes like professional fulfillment and higher screening scores for anxiety and depression using GAD-2 and PHQ-2 due to lower numbers of physicians experiencing the outcomes of interest. Logistic regression was used to evaluate the association between participant characteristics (categorical) and burnout (binary), resulting in an odds ratio. Both univariate and multivariable analyses were conducted. All models in the multivariable analyses were adjusted for age and gender as research shows these are known risk factors for burnout [19]. Only complete participant responses to OLBI were used for the burnout analyses.

Inductive thematic analysis based on the methods of Braun and Clarke was used to analyze open-text responses [20]. All open-text responses were coded and analyzed for themes by two authors (SAF, XYH), each reviewing the responses and categorizing them into themes independently. Final themes were determined through an iterative discussion process between the two authors and were validated with the larger research team. Data were suppressed where necessary to protect confidentiality and adhere to Public Health Ontario’s policies.

### Ethics

The study was approved by Public Health Ontario’s Ethics Review Board (2023.030.01). Prior to beginning the survey, participants were requested to read the consent information and provide informed consent by clicking a link before beginning the survey questionnaire. The survey became available after informed consent was indicated. During the survey, participants had the option to not answer any question. Prior to survey submission, participants were required to confirm they wished to submit their data by clicking the appropriate link.

## Results

Between April and May 2024, 454 respondents clicked the online survey link. Of these a total of 297 potential respondents were excluded for the following reasons: no consent (n = 260), not practicing public health in Canada (n = 3), no information on role to assess eligibility (n = 10), all further questions unanswered (n = 24). A total of 157 physicians met the eligibility criteria. Of these, 38 answered some questions but ended the survey prior to submission. Of the remaining 119 physicians, 118 (75.2%) completed the survey in its entirety, including the OLBI questions used for burnout analyses (S1 Fig).

### Participant and workplace characteristics

S1 Table presents the sociodemographic and workplace characteristics of physicians. Most participants identified as women (56.3%); 31% were between 40–49 years old; and 21.8% identified as a racialized person or person of colour. In terms of professional roles, 33.6% were current Medical Officers of Health, 27.7% were Associate Medical Officers of Health and 28.6% were current Public Health and Preventive Medicine residents.

S2 Table presents physicians’ perceptions of work characteristics and plans for leaving or retiring. Almost half (41.2%) of physicians were threatened, assaulted or bullied during the pandemic. When asked about psychological workplace safety, 73.1% of physicians felt safe, 21.8% did not feel safe and 5.0% preferred not to answer. Overall, 52.9% of physicians surveyed indicated that their workplace offered supports for psychological wellbeing. With regards to physical safety in the workplace, 87.4% of physicians felt safe, 10.1% did not feel safe and 2.5% preferred not to answer. Overall, 37.8% of physicians surveyed indicated that their workplace offered supports for physical wellbeing. Only 8.4% of physicians indicated their intention to retire or take another job outside of the public health sector in the next year.

### Burnout

The overall prevalence of burnout (i.e., meeting cutoffs on both the disengagement and exhaustion subscales) was 63.6%. When evaluating the subscales separately, 73.7% met the criteria for exhaustion and 72.9% met the criteria for disengagement (S3 Table). S4 Table shows participant characteristics stratified by presence or absence of burnout and the results from the logistic regression analysis exploring the association between participant characteristics and burnout measures. No significant differences were observed between any participant characteristics for burnout in the univariate analyses. After adjusting for age and gender, there were no statistically significant differences between any participant characteristics and burnout.

Both men and women experienced high levels of burnout (43% and 52%), exhaustion (40% and 54%) and disengagement (43% and 52%), respectively. Among those who met the criteria, burnout levels were highest among participants aged 30–39 years old (29%). Sub-group analyses of public health physicians (non-trainees) and residents (trainees) were conducted, and similar levels of burnout were found (55/85, 64.7% and 20/34, 58.9%, respectively).

### Burnout and secondary outcomes

S5 Table presents the prevalence of physicians who met the screening criteria for depression and anxiety using PHQ-2 and GAD-2, respectively. A minority of physicians (7.6%) exhibited symptoms of depression warranting further investigation and 19.3% of physicians met the GAD-2 screening criteria for anxiety. S6 Table shows the prevalence of moderate-to-high and low professional fulfillment and self-valuation. Most physicians (84.9%) identified as having low professional fulfillment while 15.5% experienced moderate-to-high professional fulfillment. With regards to self-valuation, 45.8% of physicians had high while 54.2% had low self-valuation.

The association of burnout and screening positive on the GAD-2 showed that physicians with burnout had 4.79 times higher odds of screening positive on the GAD-2 compared to those without burnout (95% CI 1.29 – 26.90; p-value = 0.01). Similarly, individuals with burnout were found to have 2.10 times higher odds of screening positive on the PHQ-2 screening tool compared to those without burnout (95% CI 0.38 – 21.65; p-value = 0.48). In addition, an association between burnout and professional fulfillment was observed where physicians with burnout had 12.5 times higher odds of having low professional fulfillment compared to physicians without burnout (95% CI 3.21 – 72.76; p-value <0.001).

### Summary of open-text responses

Completion of open-text fields was optional and ranged from 1 to 35 respondents depending on the question. Participants provided open-text responses to questions regarding the impacts related to their work experience during the pandemic, workplace safety, effective strategies and areas of improvement (summarized in S8 Table). Workplace harassment can be described as incivility and harassment by colleagues and supervisors (e.g., the presence of “dynamics of bullying other colleagues”) or threats from the public. Some of the physicians described threats and cyberbullying through phone calls, emails, and social media platforms. Negative comments from social media had both short- and long-term effects on physicians, who described feelings of hypervigilance, anxiety and other negative symptoms of mental well-being.

Participants described key supports to prevent or mitigate burnout, such as having a supportive team and colleagues (e.g., “I’ve felt well supported as a resident and was well integrated into the team), being physically safe in the workplace (e.g., “could work virtually if unwell or for other reason”), and recognition (e.g., “I felt supported by police and broader community” (S7 Table). They also identified areas of improvement, including poor supervision/lack of support (e.g., inappropriate involvement of certain leaders in our teams’ work led to fearful environment”), heavy workload (e.g., “required to work many hours a day 7 days a week…”), workplace harassment and intimidation, lack of mental health resources within their organization, lack of workplace safety measures (e.g., “First building was not well ventilated, high density of people working during first wave of COVID”), and compensation (e.g., “some lieu time, but hundreds of hours not compensated”) (S8 Table).

### Workplace harassment

A minority of physicians expanded on their experiences of workplace harassment. Those who responded reported being bullied or intimidated at work, mostly through verbal threats. When it occurred, these behaviors came from both colleagues and external sources, such as members of the public.

### Workplace safety

Many physicians provided additional information and reported feeling unsafe at work due to lack of appropriate supervision or support within their team, heavy workload, workplace intimidation, lack of mental health support, or lack of physical safety measures.

### Opinions on strategies to prevent burnout

Participants were invited to share their ideas on how to prevent burnout. Responses were classified into the following themes: a) personal strategies, b) interpersonal strategies, c) organizational supports, d) workplace safety, and e) organizational culture. Under personalized self-care, physicians shared common themes such as time management, maintaining work-life balance, and adopting a healthy lifestyle. Regarding interpersonal strategies, physicians commented on the positive support they received from peers and colleagues. Participants also described the positive impacts of supportive programs and group discussions within their organization. They also recommended specific strategies, such as adequate compensation, yoga, wellness newsletters, and meditation sessions. In discussing workplace safety, physicians highlighted the importance of implementing workplace safety measures, such as remote work options, guidelines to address workplace harassment and intimidation, and workload management and monitoring for continuous improvement. Lastly, physicians encouraged building a positive organizational culture with the following recommendations: setting SMART (specific, measurable, achievable, relevant, time-bound) goals and clear expectations, highlighting the importance of psychological safety, and collaboration.

## Discussion

### Prevalence of burnout

This cross-sectional study explored the prevalence of burnout, associations between participant workplace characteristics and burnout, and its associated secondary outcomes among Canadian public health physician leaders and residents one year after the end of the COVID-19 pandemic. Overall, 64% met the cutoff for burnout with 74% and 73% meeting cutoffs for exhaustion and disengagement subscales, respectively.

Our findings fall within the range identified through previous studies in the public health and healthcare workforces. Our previous cross-sectional study found the prevalence of burnout in the public health workforce was 78.7% [10]. Another cross-sectional study of the public health workforce in Canada found the prevalence of burnout to be 81% in the first wave of the pandemic (2020) and 64% in late 2022-early 2023 [9]. The only pre-pandemic study of burnout in the public health workforce showed a 50% prevalence in China [21]. While it is promising to see that Canadian public health physicians have experienced an improvement in the prevalence of burnout over the course of a year, a prevalence of 64% remains concerning. Additionally, a study on healthcare sector workers in Canada showed 33% felt “exhausted and burnt-out” “most of the time/always”. This prevalence of burnout in the Canadian healthcare workforce is lower than what we found in public health physicians, suggesting an opportunity for improvement [22].

These findings are concerning given that other studies have found that burnout was associated with greater odds of intending to leave public health or retire earlier than anticipated [9,10], posing a serious threat to the public health system’s ability to effectively provide essential services and respond to future emerging threats.

### Associations between participant characteristics and burnout

No statistically significant associations were found in univariate or multivariable logistic regression. Trends were seen for increasing odds of burnout: increasing age (60 + years AOR 3.44, CI 95% 0.67-17.70), 26 or more years of work experience (AOR 6.07, CI 95% 0.26-141.33), and identifying as racialized or a person of colour (OR 1.78, CI 95% 0.64-4.94). As well, trends were seen for decreasing odds of burnout: identifying as a woman (AOR 0.61, CI 95% 0.26-1.44) and being a caregiver for children under 18 years (AOR 0.74, CI 95% 0.27-2.03). In a systematic review exploring predictors of burnout among US healthcare providers, the authors found that women may be more likely to report burnout, but most studies did not find an association with gender (73 studies); younger participants may be more likely to report burnout (53 studies); ethnicity was unlikely to be associated with burnout (17 studies); and having children was likely not associated with having burnout (29 studies) [23]. However, the quality rating for studies was low due to inconsistency of effects. Further study is needed to better understand whether demographic characteristics are associated with burnout to inform prevention and early intervention strategies.

### Associations between burnout and sequelae

This study also examined the associations between burnout and other potential negative outcomes. We found that 7.6% of physicians exhibited symptoms of depression using PHQ-2 and 19.3% of physicians met the GAD-2 screening criteria for anxiety. We found an association between burnout and screening positive for anxiety, as physicians with burnout were found to have 4.79 (CI 95% 1.29-26.9, p = 0.01) times higher odds of screening positive on the GAD-2 compared to those without burnout. Similarly, individuals with burnout were found to have 2.10 (CI 95% 0.38-21.65, p = 0.48) times higher odds of screening positive on the PHQ-2 screening tool compared to those without burnout, although this was not statistically significant. It is important to note that the purpose of using the PHQ-2 and GAD-2 is to screen for symptoms of depression and anxiety, not diagnose, and must be interpreted in the context of other clinical findings. Our findings are similar to other studies that have also shown a relationship between depression, anxiety, and occupational burnout [9,24–26]. Furthermore, we also examined the association between burnout and professional fulfillment. A vast majority of physicians indicated low professional fulfilment and those with burnout were found to have 12.5 (CI 95% 3.21-72.76, p < 0.001) times higher odds of having low professional fulfillment compared to physicians without burnout. Our results are similar to other studies that also found physicians experiencing burnout reported poorer professional fulfillment [27,28]. Job dissatisfaction is both a consequence and predictor of burnout and understanding the associations between burnout and secondary outcomes such as depression, anxiety, and professional fulfillment is crucial for developing effective workplace interventions in public health settings.

### Participants’ opinions on contributors and mitigators of burnout

Our study also captured the opinions of public health physicians on factors that contributed to and mitigated burnout. Contributing factors included workplace harassment, negative comments from social media, lack of support within the organization, lack of workplace safety measures, and heavy workload and pressures. This is consistent with results from a previous systematic review which also found that burnout may be associated with workload and job stress (56 studies); that having stronger leadership is associated with less burnout (20 studies); and that inadequate work/life balance is associated with more burnout reporting (31 studies) [23]. However, there was inconsistency in effects found in all of these domains and low-moderate quality evidence) pointing to a need for further study of how workplace factors are associated with burnout.

Respondents felt that several strategies could mitigate burnout including building resilience through self-care, relationships with others, and being in an organization that is both physically and psychologically safe from internal and external threats. Currently, there is limited research on how to prevent and mitigate burnout in the public health physician workforce. Drawing from the broader literature, Haslam *et al*. conducted a systematic review and meta-analysis of randomized trials testing interventions to reduce physician burnout [29]. Interventions included coaching/counselling, education on stress reduction/coping strategies, discussion groups, mindfulness activities (meditation, yoga), schedule change, and one drug (cannabinol). Some improvements were seen in the domains of emotional exhaustion, depersonalization, and personal accomplishment, but the authors did not feel these would result in meaningful clinical changes. Significant further research is needed on effective interventions to prevent and mitigate burnout in physicians. The factors contributing to and mitigating burnout identified by our study participants may serve as hypotheses for future research.

### Research priorities

Future research priorities should include understanding the long-term outcomes and recovery from burnout. The current literature is cross-sectional in nature, and the field would benefit from study designs that follow the trajectory of physicians with burnout over time. Further research would also benefit from a deeper understanding of the trends in associations between participant characteristics and burnout, particularly the experience of racialized people and people of colour, and if having children under 18 years is protective. Lastly, developing evidence-based supports for early career public health physicians given their higher rates of burnout and developing evidence-based prevention measures that are feasible to implement prior to and during a public health emergency are needed.

### Study limitations

There were several limitations to our study. Given its cross-sectional design, this study cannot conclude causal relationships between burnout and secondary outcomes like depression, anxiety, and professional fulfillment. We also did not assess co-morbidities, such as other mental health diagnoses. Distribution of the survey through public health associations and networks may have introduced selection bias, as individuals with burnout who left or retired prior to the release of our questionnaire would not be included. This may have led to an underestimation of burnout prevalence as well as limiting generalizability of the findings to the wider public health physician workforce. All measures were self-reported and susceptible to response and recall biases.

## Conclusions

In summary, burnout among public health physicians and residents requires attention to improve the well-being of this group, as burnout levels remained high in 2024, which is a year after the official end of the pandemic. This group of professionals often serve in formal and informal leadership roles within their organization and can have significant impacts on workplace culture. Unaddressed burnout and its consequences in public health physician leaders have the potential to negatively impact recruitment and retention in the field of public health along with broader organizational and public health system effectiveness. Workplace culture, adequate policies, supportive working environments, and adequate resources can all contribute positively to public health physicians’ and residents’ resilience throughout their career.

## Supporting information

S1 Fig Flow diagram of study participants. (TIF)

S1 Table Sociodemographic and workplace characteristics of survey physicians (n = 119). (DOCX)

S2 Table Summary of physicians’ perceptions of work characteristics and plans (n = 119). (DOCX)

S3 Table Burnout, Exhaustion and Disengagement prevalence and mean scores (n = 118). (DOCX)

S4 Table Participant characteristics by burnout status and associations with burnout from univariate and multivariable logistic regression (n = 118). (DOCX)

S5 Table Prevalence of screening positive (3 or more) for Generalized Anxiety Disorder and Depression using screening tools (n = 119). (DOCX)

S6 Table Prevalence of Stanford well-being measures. (DOCX)

S7 Table Supports to prevent or mitigate burnout as described in open-text responses (n = number of responses*). (DOCX)

S8 Table Areas for improvement to prevent or mitigate burnout as described in open-text responses (n = number of responses*). (DOCX)

S1 Appendix Checklist for Reporting Results of Internet E-Surveys (CHERRIES). (DOCX)

S2 Appendix HaPPHIER: Healing Physicians in Public Health for an Inclusive and Equitable Recovery Survey. (DOCX)

## Abbreviations

AOR: Adjusted odds ratio
CI: Confidence interval
GAD-2: Generalized Anxiety Disorder 2-item
OLBI: Oldenburg Burnout Inventory
OR: Odds ratio
PHQ-2: Patient Health Questionnaire-2
SPFI: Stanford Professional Fulfillment Index.
