# Supplementary material for: Burnout among public health physicians and residents in Canada following the COVID-19 pandemic: A cross-sectional study
Source: PLOS Ment Health. 2025 Dec 23;2(12):e0000527. doi: 10.1371/journal.pmen.0000527 (PMC12798441; doi:10.1371/journal.pmen.0000527)
Supplement: S1 Table — (DOCX) [file pmen.0000527.s002.docx]

**S1 Table.** Sociodemographic and workplace characteristics of survey physicians (n = 119)

| **Characteristics** | **n** | **%** |
| --- | --- | --- |
| **Participant Demographics** | | |
| **Age (years)** |  |  |
| 20-29 years old | 15 | 12.6% |
| 30-39 years old | 34 | 28.6% |
| 40-49 years old | 37 | 31.1% |
| 50-59 years old | 9 | 7.6% |
| 60-64 years old | 11 | 9.2% |
| 65 years old or older | 8 | 6.7% |
| Prefer not to answer | 5 | 4.2% |
| **Gender** |  |  |
| Woman | 67 | 56.3% |
| Man | 45 | 37.8% |
| Other/non-binary | 3 | 2.5% |
| Prefer not to answer | 4 | 3.4% |
| **Racialized Person or Person of Colour** |  |  |
| Yes | 26 | 21.8% |
| No | 86 | 72.3% |
| Prefer not to answer | 7 | 5.9% |
| **Total Household Income in 2023 (CDN)** |  |  |
| Less than $150,000 | 24 | 20.2% |
| $150,000 to $249,999 | 15 | 12.6% |
| $250,000 to $349,999 | 37 | 31.1% |
| $350,000 to $449,999 | 20 | 16.8% |
| More than $450,000 | 16 | 13.4% |
| Prefer not to answer | 7 | 5.9% |
| **Caregiver for Children < 18 years** |  |  |
| Yes | 48 | 40.3% |
| No | 70 | 58.8% |
| Prefer not to answer | 1 | 0.8% |
| **Caregiver for Adult Dependents** |  |  |
| Yes | 13 | 10.9% |
| No | 104 | 87.4% |
| Prefer not to answer | 2 | 1.7% |
| **Employment Status** |  |  |
| Employed or resident full-time | 109 | 91.6% |
| Employed or resident part-time | 3 | 2.5% |
| Other | 5 | 4.2% |
| Prefer not to answer | 2 | 1.7% |
| **Years Worked in Public Health^*^** |  |  |
| Less than or equal to 5 years | 21 | 23.9% |
| 6 to 15 years | 36 | 40.9% |
| 16 to 25 years | 19 | 21.6% |
| 26 years or more | 11 | 12.5% |
| Missing | 1 | 1.1% |
| **Current Role** |  |  |
| Medical Officer of Health/Medical Health Officer or equivalent | 40 | 33.6% |
| Associate Medical Officer of Health/Medical Health Officer or equivalent | 33 | 27.7% |
| Physician Leader and CEO of a public health unit/regional health authority | 7 | 5.9% |
| Public Health and Preventive Medicine Residency Program Director/Assistant Program Director or equivalent | 5 | 4.2% |
| Public Health and Preventive Medicine Resident | 34 | 28.6% |
| **Workplace Characteristics** | | |
| **Work or Training Setting** |  |  |
| In office, clinic or community setting | 14 | 11.8% |
| Virtual | 26 | 21.8% |
| Hybrid - both virtual and in-person settings | 77 | 64.7% |
| Missing | 2 | 1.7% |
| **Type of Jurisdiction Served by Public Health Agency** |  |  |
| Urban (in the city) | 48 | 40.3% |
| Rural (in the country) | 10 | 8.4% |
| Mixed (includes urban and rural areas) | 61 | 51.3% |
| **Northern/Remote Jurisdiction** |  |  |
| Yes | 10 | 8.4% |
| No | 108 | 90.8% |
| Prefer not to answer | 1 | 0.8% |
| **Population Size of Jurisdiction** |  |  |
| Less than or equal to 100k | 7 | 5.9% |
| 101k to 500k | 31 | 26.1% |
| 501k to 2 million | 54 | 45.4% |
| 3 million or more | 10 | 8.4% |
| Missing | 17 | 14.3% |

*Years worked in public health exclude residency training; residents not included in total.
