# Supplementary material for: Burnout among public health physicians and residents in Canada following the COVID-19 pandemic: A cross-sectional study
Source: PLOS Ment Health. 2025 Dec 23;2(12):e0000527. doi: 10.1371/journal.pmen.0000527 (PMC12798441; doi:10.1371/journal.pmen.0000527)
Supplement: S2 Table — (DOCX) [file pmen.0000527.s003.docx]

**S2 Table**. Summary of physicians’ perceptions of work characteristics and plans (n = 119)

| **Characteristics** | **n** | **%** |
| --- | --- | --- |
| **Threatened, Assaulted or Bullied during the pandemic** |  |  |
| Yes | 49 | 41.2% |
| No | 68 | 57.1% |
| Prefer not to answer | 2 | 1.7% |
| **Felt Psychologically Safe at Workplace** |  |  |
| Yes | 87 | 73.1% |
| No | 26 | 21.8% |
| Prefer not answer/Missing | 6 | 5.0% |
| **Workplace Supports for Psychological Wellbeing** |  |  |
| Yes | 63 | 52.9% |
| No | 49 | 41.2% |
| Prefer not to answer | 7 | 5.9% |
| **Felt Physically Safe at Workplace** |  |  |
| Yes | 104 | 87.4% |
| No | 12 | 10.1% |
| Prefer not to answer | 3 | 2.5% |
| **Workplace Supports for Physical Wellbeing** |  |  |
| Yes | 45 | 37.8% |
| No | 70 | 58.8% |
| Prefer not to answer/missing | 4 | 3.4% |
| **Intention to Leave or Retire Within the Next Year** |  |  |
| No | 89 | 74.8% |
| Yes, to retire | 4 | 3.4% |
| Yes, to take another job in public health | 10 | 8.4% |
| Yes, to take another job not in public health | 6 | 5.0% |
| Other | 5 | 4.2% |
| Prefer not to answer/missing | 5 | 4.2% |
