# Supplementary material for: Burnout among public health physicians and residents in Canada following the COVID-19 pandemic: A cross-sectional study
Source: PLOS Ment Health. 2025 Dec 23;2(12):e0000527. doi: 10.1371/journal.pmen.0000527 (PMC12798441; doi:10.1371/journal.pmen.0000527)
Supplement: S3 Table — (DOCX) [file pmen.0000527.s004.docx]

**S3 Table**. Burnout, Exhaustion and Disengagement prevalence and mean scores (n = 118)

| **OLBI Outcomes** | **Survey physicians** | | | | | | |
| --- | --- | --- | --- | --- | --- | --- | --- |
|  | **Yes** | | **No** | | **Mean** | **95% CI** | |
|  | ***n*** | **%** | ***n*** | **%** |  |  |  |
| Burnout | 75 | 63.6 | 43 | 36.4 | 2.43 | 2.34 | 2.51 |
| Exhaustion | 87 | 73.7 | 31 | 26.3 | 2.53 | 2.43 | 2.63 |
| Disengagement | 86 | 72.9 | 32 | 27.1 | 2.33 | 2.23 | 2.42 |

OLBI: Oldenburg Burnout Inventory; CI: Confidence Interval
