# Supplementary material for: Burnout among public health physicians and residents in Canada following the COVID-19 pandemic: A cross-sectional study
Source: PLOS Ment Health. 2025 Dec 23;2(12):e0000527. doi: 10.1371/journal.pmen.0000527 (PMC12798441; doi:10.1371/journal.pmen.0000527)
Supplement: S4 Table — (DOCX) [file pmen.0000527.s005.docx]

**S4 Table.** Participant characteristics by burnout status and associations with burnout from univariate and multivariable logistic regression (n = 118).

| **Characteristic** | **Burnout**  **n (%)** | **No Burnout**  **n (%)** | **Unadjusted OR**  **(95% CI)** | **Adjusted* OR**  **(95% CI)** |
| --- | --- | --- | --- | --- |
| **Age (years)** |  |  |  |  |
| 20-29 years old | 8 (10.7) | 7 (16.3) | Ref | Ref |
| 30-39 years old | 22 (29.3) | 11 (25.6) | 1.75 (0.50 – 6.08) | 1.58 (0.44 – 5.61) |
| 40-49 years old | 21 (28.0) | 16 (37.2) | 1.15 (0.34 – 3.83) | 1.21 (0.36 – 4.08) |
| 50-59 years old | 5 (6.7) | 4 (9.3) | 1.09 (0.21 – 5.76) | 1.12 (0.21 – 5.94) |
| 60+ | 14 (18.7) | 5 (11.6) | 2.45 (0.58 – 10.33) | 3.44 (0.67 – 17.70) |
| Prefer not to answer | 5 (6.7) | 0 (0.0) | – | – |
| **Gender** |  |  |  |  |
| Man | 32 (42.7) | 12 (27.9) | Ref | Ref |
| Woman | 39 (52.0) | 28 (65.1) | 0.52 (0.23 – 1.19) | 0.61 (0.26 – 1.44) |
| Other/non-binary | 0 (0.0) | 3 (7.0) | – | – |
| Prefer not to answer | 4 (5.3) | 0 (0.0) | – | – |
| **Work experience (years)^†^** |  |  |  |  |
| < 5 years | 14 (18.7) | 7 (16.3) | Ref | Ref |
| 6 to 15 years | 22 (29.3) | 14 (32.6) | 0.79 (0.25 – 2.43) | 0.98 (0.26 – 3.63) |
| 16 to 25 years | 11 (14.7) | 8 (18.6) | 0.69 (0.19 – 2.49) | 1.02 (0.17 – 6.15) |
| 26 years or more | 10 (13.3) | 1 (2.3) | 5.00 (0.53 – 47.29) | 6.07 (0.26 – 141.33) |
| Missing | 1 (1.3) | 0 (0.0) | – | – |
| **Caregiver for Children < 18 years** |  |  |  |  |
| No | 45 (60.0) | 24 (55.8) | Ref | Ref |
| Yes | 29 (38.7) | 19 (44.2) | 0.81 (0.38 – 1.74) | 0.74 (0.27 – 2.03) |
| Prefer not to answer | 1 (1.3) | 0 (0.0) | – | – |
| **Racialized or person of colour** |  |  |  |  |
| No | 55 (46.6) | 31 (26.3) | Ref | Ref†† |
| Yes | 19 (16.1) | 6 (5.1) | 1.78 (0.64 – 4.94) | - |
| Prefer not to answer | 6 (5.1) | 1 (0.1) | - | - |

OR: Odds Ratio
*Adjusted for age and gender

†Years worked in public health exclude residency training; residents not included in total

††Not reported for confidentiality
