# Supplementary material for: Burnout among public health physicians and residents in Canada following the COVID-19 pandemic: A cross-sectional study
Source: PLOS Ment Health. 2025 Dec 23;2(12):e0000527. doi: 10.1371/journal.pmen.0000527 (PMC12798441; doi:10.1371/journal.pmen.0000527)
Supplement: S5 Table — (DOCX) [file pmen.0000527.s006.docx]

**S5 Table.** Prevalence of screening positive (3 or more) for Generalized Anxiety Disorder and Depression using screening tools (n = 119)

| **Outcomes** | **Survey physicians** | | | | | | |
| --- | --- | --- | --- | --- | --- | --- | --- |
|  | **Score > 3** | | **Score < 3** | | **Mean** | **95% CI** | |
|  | ***n*** | **%** | ***n*** | **%** |  |  |  |
| Depression (PHQ-2) | 9 | 7.6 | 110 | 92.4 | 0.98 | 0.74 | 1.22 |
| Anxiety (GAD-2) | 23 | 19.3 | 96 | 80.7 | 1.50 | 1.22 | 1.77 |
