# Supplementary material for: Burnout among public health physicians and residents in Canada following the COVID-19 pandemic: A cross-sectional study
Source: PLOS Ment Health. 2025 Dec 23;2(12):e0000527. doi: 10.1371/journal.pmen.0000527 (PMC12798441; doi:10.1371/journal.pmen.0000527)
Supplement: S6 Table — (DOCX) [file pmen.0000527.s007.docx]

**S6 Table.** Prevalence of Stanford well-being measures

| **Outcomes** | **Survey physicians** | | | | | | |
| --- | --- | --- | --- | --- | --- | --- | --- |
|  | **High** | | **Low** | | **Mean** | **95% CI** | |
|  | ***n*** | **%** | ***n*** | **%** |  |  |  |
| Professional Fulfillment Index (n=119) | 18 | 15.1 | 101 | 84.9 | 2.16 | 2.00 | 2.31 |
| Self-Valuation / Self-Compassion (n=118) | 54 | 45.8 | 64 | 54.2 | 1.94 | 1.78 | 2.10 |
