# Supplementary material for: Burnout among public health physicians and residents in Canada following the COVID-19 pandemic: A cross-sectional study
Source: PLOS Ment Health. 2025 Dec 23;2(12):e0000527. doi: 10.1371/journal.pmen.0000527 (PMC12798441; doi:10.1371/journal.pmen.0000527)
Supplement: S7 Table — (DOCX) [file pmen.0000527.s008.docx]

**S7 Table**. Supports to prevent or mitigate burnout as described in open-text responses (n=number of responses*)

| **Category** | **Sub-categories** | **Illustrative comments** |
| --- | --- | --- |
| **Organizational culture (n=54)** | Workplace psychological safety (clear goals and expectations, recognition, transparency, resource availability) | “I’ve felt well supported as a resident and was well integrated into the team. I was given more responsibilities than Public Health and Preventive Medicine residents prior to the COVID-19 pandemic”  “Lots of support from senior leadership team. We were in this together” |
|  | Teamwork and collaboration | “Working with others to achieve a common good.”  *“Solidarity and collaboration”* |
|  | Collegiality | “Colleagues and believing in what we do” |
| **Workplace safety (n=60)** | Physical security | “IPAC measures were in place”  “The normal mechanisms were in place: ID card-activated doors” |
|  | Ergonomics | *“Ergonomic tips for the virtual office”* |
|  | Virtual option | “could work virtually if unwell or for other reason” |
| **Organizational supports (n=56)** | Structured workplace wellness programs, including informational resources | “Employee and Family Assistance Program, extra coverage for psychologist/therapist, supportive colleagues, wellness tips…”  “Some were useful (group debriefs) some less so (e.g. Zoom meditation session during busy meeting times)” |
|  | Counselling | “Covered by the Quebec Physician Assistance Program” |
| **Interpersonal strategies (n=21)** | Community supports (e.g., positive recognition) | “I felt supported by police and broader community” |
|  | Social supports (e.g., work-life balance, friends, family, pets) | “friends, family, new pets, supportive local doctors”  “spending time with spouse & kids” |
| **Personal strategies (n=54)** | Hobbies and activities | “Reading, running, singing, swimming” |
|  | Motivation (e.g., sense of accomplishment) | *“Indispensable work to protect the population”*  “Doing meaningful work and bringing hope” |

*Italicized comments* are expansions of acronyms, spelling/grammar corrections or translated from French.

*Participants could contribute more than once in each category
