# Supplementary material for: Burnout among public health physicians and residents in Canada following the COVID-19 pandemic: A cross-sectional study
Source: PLOS Ment Health. 2025 Dec 23;2(12):e0000527. doi: 10.1371/journal.pmen.0000527 (PMC12798441; doi:10.1371/journal.pmen.0000527)
Supplement: S8 Table — (DOCX) [file pmen.0000527.s009.docx]

**S8 Table.** Areas for improvement to prevent or mitigate burnout as described in open-text responses (n=number of responses*)

| **Category** | **Sub-categories** | **Illustrative comments** |
| --- | --- | --- |
| **Poor work supervision and management (n=19)** | - Lack of supervision - Lack of support and resources - Heavy workload | “inappropriate involvement of certain leaders in our teams’ work led to fearful environment”  “required to work many hours a day 7 days a week no compensation or respite” |
| **Lack of physical safety measures in the workplace (n=9)** | - Lack of physical security measures - Lack of IPAC measures | “It would easily be known that all the *staff* were at the single site, and we had very strong anti-*public health* personalities who entrenched themselves in our community (even from outside the province) given they felt it was a safe haven for them to do what they intended to do”  “We switched buildings during summer 2021. First building was not well ventilated, high density of people working during first wave of COVID” |
| **Workplace harassment and intimidation (n=3)** |  | “*Dynamics of bullying other colleagues*” |
| **Lack of mental health support within the organization (n=20)** | - Lack of awareness | “Sadly, decisions in the organization did not take into account the wellbeing of workers. No interventions were made in that spirit”  “workplace actively encouraged unsafe work conditions. No supports for wellbeing” |
| **Inadequate compensation (n=7)** | - Lack of overtime payment or time in lieu | “Some lieu time, but hundreds of hours not compensated” |

*Italicized comments* are expansions of acronyms, spelling/grammar corrections or translated from French.

*Participants could contribute more than once in each category
