# Supplementary material for: Burnout among public health physicians and residents in Canada following the COVID-19 pandemic: A cross-sectional study
Source: PLOS Ment Health. 2025 Dec 23;2(12):e0000527. doi: 10.1371/journal.pmen.0000527 (PMC12798441; doi:10.1371/journal.pmen.0000527)
Supplement: S1 Appendix — (DOCX) [file pmen.0000527.s010.docx]

**S1 Appendix.** Checklist for Reporting Results of Internet E-Surveys (CHERRIES)

| **Checklist item** | **Description** |
| --- | --- |
| **Design** | |
| Survey design | Cross-sectional, convenience sample |
| **Institutional Review Board approval and informed consent process** | |
| Institutional Review Board approval | Yes (Public Health Ontario Ethics Review Board) |
| Informed consent | Voluntary informed consent was obtained from all participants prior to completing the survey. This included length of the survey, data storage and retention policies, and purpose of the study. |
| Data protection | Participants were informed of the legislation under which data were collected and stored, along with safeguards to protect the information collected. |
| **Development and pre-testing** | |
| Development and testing | The survey was developed based on our previous survey [10] and with additional validated measurement tools. The survey technology is used regularly by Public Health Ontario (Surveys@PHO) and specific functionality of this survey was tested amongst the research team. |
| **Recruitment process and description of the sample having access to the questionnaire** | |
| Open vs. closed survey | Open survey |
| Contact mode | Electronic via email |
| Advertising the survey | Physicians were invited to participate through email distribution to various professional distribution lists |
| **Survey administration** | |
| Web/email | Survey was hosted on Surveys@PHO which is a web-based survey platform |
| Context | The survey was only accessible through the link sent through the email invitation and not searchable online. |
| Mandatory/voluntary | Voluntary |
| Incentives | None |
| Time/date | Data collection occurred from April 8-May 31, 2024 |
| Randomization of items or questionnaires | Not randomized |
| Adaptive questioning | Simple logic was used for some questions that had sub-questions. This reduced the number of questions that were not applicable to some participants. |
| Number of items | 68 |
| Number of screens | Not tracked |
| Completeness check | All questions except open text responses required an answer. “Prefer not to answer” was always an option. |
| Review step | Participants were able to navigate back through the survey and were required to click a submit button at the end. |
| **Response rates** | |
| Unique site visitor | Not tracked (unique codes not used and IP address not feasible as some organizations use a common IP and this would prevent multiple respondents from the same organization) |
| View rate | Not tracked |
| Participation rate | Tracked (see Fig 1) |
| Completion rate | Tracked (see Fig 1) |
| **Preventing multiple entries from the same individual** | |
| Cookies used | Not applicable |
| IP check | Not applicable. Organizations often use a common IP address. Some organizations had the potential for multiple respondents, so we elected not to use an IP check |
| Log file analysis | Not applicable |
| Registration | Not applicable |
| **Analysis** | |
| Handling of incomplete questionnaires | Analyses were conducted for completed questionnaires based on needed data (see Fig 1) |
| Questionnaires submitted with an atypical timestamp | The survey was open for pre-specified dates. No questionnaires were submitted outside of the timeframe. |
| Statistical correction | Not conducted. We have estimated the number of public health physicians in Canada based on publicly available information, but do not have adequate information to understand if the sample is representative. |
