# Supplementary material for: Burnout among public health physicians and residents in Canada following the COVID-19 pandemic: A cross-sectional study
Source: PLOS Ment Health. 2025 Dec 23;2(12):e0000527. doi: 10.1371/journal.pmen.0000527 (PMC12798441; doi:10.1371/journal.pmen.0000527)
Supplement: S2 Appendix — (DOCX) [file pmen.0000527.s011.docx]

**S2 Appendix.** HaPPHIER: Healing Physicians in Public Health for an Inclusive and Equitable Recovery Survey

Work Characteristics

1. Do you currently practice public health in Canada?

- Yes
- No (if selected end survey)

1. What best describes your current role?

- Medical Officer of Health/Medical Health Officer or equivalent
- Associate Medical Officer of Health/Associate Medical Health Officer or equivalent
- Physician Leader and CEO of a public health unit/regional health authority
- Public Health and Preventive Medicine Residency Program Director/Assistant Program Director or equivalent
- Public Health and Preventive Medicine Resident
- None of the above (if selected end survey)

1. Did you hold another role between January 2020 and January 2023?

- Yes
  - Medical Officer of Health/Medical Health Officer or equivalent
  - Associate Medical Officer of Health/Associate Medical Health Officer or equivalent
  - Physician Leader and CEO of a public health unit/regional health authority
  - Public Health and Preventive Medicine Residency Program Director/Assistant Program Director or equivalent
  - Public Health and Preventive Medicine Resident
  - Other [description]
- No
- Prefer not to answer

1. How many years have you worked in public health (local, provincial or federal) excluding residency training?

- None, I am currently a resident
- [xx] years
- Prefer not to answer

1. What best describes the jurisdiction your public health agency mainly (>50%) serves?

- Urban (in the city)
- Rural (in the country)
- Mixed (includes urban and rural areas)
- Prefer not to answer

1. Is your jurisdiction northern or remote?

*Northern is defined as anything north of the 60th parallel.*

*Remote is defined as a geographical area where a community is located over 350 km from the nearest service centre having year-round access by land and/or water routes normally used in all weather conditions, fly-in communities, and those not accessible year-round and in all weather conditions.*

- Yes
- No
- Prefer not to answer

1. What is the approximate population size of the jurisdiction your public health agency serves?

- [xx] people
- Prefer not to answer

1. What was your employment status (>50% of the time) during the pandemic (i.e., between 2020 and present)?

- Employed or resident Full-time
- Employed or resident Part-time
- On leave of absence
- Other [text]
- Prefer not to answer

1. What was/is your usual work or training setting during the pandemic (i.e., between 2020 and present)?

- In office, clinic or community setting
- Virtual
- Hybrid - both virtual and in-person settings
- Prefer not to answer

1. Are you considering leaving your organization within the next year? If so, what are you planning to do?

- No
- Yes, to retire
- Yes, to pursue further education
- Yes, to take another job in public health
- Yes, to take another job not in public health
- Other [text]
- Prefer not to answer

1. *[10a - Display if selected any “yes” in Q10 to leaving your organization within the next year]* For approximately how long have you been considering leaving your organization?

- Less than 3 months
- 3-6 months
- 6-18 months
- Prior to March 2020
- Prefer not to answer

1. *[Q10b - Display if selected another job in Q10]* Have you recently taken any steps towards leaving your organization, such as applying or interviewing for a new position outside of your organization?

- Yes
- No
- Prefer not to answer

1. *[Q10c - Display if selected yes to retire in Q10]* Have you recently taken any steps towards retiring, such as meeting with HR or submitting relevant paperwork?

- Yes
- No
- Prefer not to answer

1. *[Q10d - Display if selected yes to any in Q10]* Did the COVID-19 pandemic impact your decision to stay or leave your organization?

- I was thinking about staying, but COVID made me want to leave
- I was thinking about staying, and COVID made me want to stay more
- I was thinking about leaving, but COVID made me want to stay
- I was thinking about leaving, but COVID made me want to leave more
- COVID did not impact my decision to leave or stay
- Prefer not to answer

1. Have you been threatened, assaulted, or bullied because of your work during the pandemic?

- Yes
- No
- Prefer not to answer

*If yes, please describe briefly. (text box- optional question- max 50 characters)*

1. *[Q15b - Display if selected yes to Q15 threatened/assaulted/bullied]* Was police action taken in response to being threatened or assaulted because of your work during the pandemic? Police action could involve warnings or fines.

- Yes [*description*]
- No
- Prefer not to answer

1. *[Q15c - Display if selected yes to Q15 threatened/assaulted/bullied]* Does being threatened, assaulted, or bullied because of your work during the pandemic continue to impact you?

- Yes
- No
- Prefer not to answer

*If yes/no, please describe briefly. (text box- optional question- max 50 characters)*

1. Did you feel psychologically safe in your workplace during the COVID-19 pandemic?

- Yes
- No
- Prefer not to answer

*If Yes or No, please describe briefly. (text box- optional question – max 50 characters).*

1. Did your workplace offer supports for your psychological wellbeing during COVID-19?

- Yes
- No
- Prefer not to answer

*If Yes or No, please describe briefly. (text box- optional question – max 50 characters).*

1. Did you feel physically safe in your workplace during the COVID-19 pandemic?

- Yes
- No
- Prefer not to answer

*If Yes or No, please describe briefly. (text box- optional question – max 50 characters).*

1. Did your workplace offer supports for your physical wellbeing during COVID-19?

- Yes
- No
- Prefer not to answer

*If yes, please describe briefly. (text box – optional question – max 50 characters)*

Oldenburg Burnout Inventory

|  | Strongly agree | Agree | Disagree | Strongly disagree |
| --- | --- | --- | --- | --- |
| 1. I always find new and interesting aspects in my work | 1 | 2 | 3 | 4 |
| 1. There are days when I feel tired before I arrive at work | 4 | 3 | 2 | 1 |
| 1. It happens more and more often that I talk about my work in a negative way | 4 | 3 | 2 | 1 |
| 1. After work, I tend to need more time than in the past in order to relax and feel better | 4 | 3 | 2 | 1 |
| 1. I can tolerate the pressure of my work very well | 1 | 2 | 3 | 4 |
| 1. Lately, I tend to think less at work and do my job almost mechanically | 4 | 3 | 2 | 1 |
| 1. I find my work to be a positive challenge | 1 | 2 | 3 | 4 |
| 1. During my work, I often feel emotionally drained | 4 | 3 | 2 | 1 |
| 1. Over time, one can become disconnected from this type of work | 4 | 3 | 2 | 1 |
| 1. After working, I have enough energy for my leisure activities | 1 | 2 | 3 | 4 |
| 1. Sometimes I feel sickened by my work tasks | 4 | 3 | 2 | 1 |
| 1. After my work, I usually feel worn out and weary | 4 | 3 | 2 | 1 |
| 1. This is the only type of work that I can imagine myself doing | 1 | 2 | 3 | 4 |
| 1. Usually, I can manage the amount of my work | 1 | 2 | 3 | 4 |
| 1. I feel more and more engaged in my work | 1 | 2 | 3 | 4 |
| 1. When I work, I usually feel energized | 1 | 2 | 3 | 4 |

Patient Health Questionnaire-2 and General Anxiety Disorder-2

Over the last 2 weeks, how often have you been bothered by the following problems?

|  | Not at all | Several days | More than half the days | Nearly every day |
| --- | --- | --- | --- | --- |
| 1. Little interest or pleasure in doing things | 0 | 1 | 2 | 3 |
| 1. Feeling down, depressed or hopeless | 0 | 1 | 2 | 3 |
| 1. Feeling nervous, anxious or on edge | 0 | 1 | 2 | 3 |
| 1. Not being able to stop or control worrying | 0 | 1 | 2 | 3 |

Stanford Professional Fulfillment Index

How true do you feel the following statements are about you during the past 2 weeks?

|  | Not true at all | Somewhat true | Moderately true | Very true | Completely true |
| --- | --- | --- | --- | --- | --- |
| 1. I feel happy at work | 0 | 1 | 2 | 3 | 4 |
| 1. I feel worthwhile at work | 0 | 1 | 2 | 3 | 4 |
| 1. My work is satisfying to me | 0 | 1 | 2 | 3 | 4 |
| 1. I feel in control when dealing with difficult problems at work | 0 | 1 | 2 | 3 | 4 |
| 1. My work is meaningful to me | 0 | 1 | 2 | 3 | 4 |
| 1. I’m contributing professionally (e.g., teaching, research, and leadership) in the ways I value most | 0 | 1 | 2 | 3 | 4 |

Stanford Self-Valuation / Self-Compassion Index

How true do you feel the following statements are about you during the past 2 weeks?

|  | Never | Rarely | Sometimes | Often | Always |
| --- | --- | --- | --- | --- | --- |
| 1. When I made a mistake, I felt more self-condemnation than self-encouragement to learn from the experience | 0 | 1 | 2 | 3 | 4 |
| 1. I was less compassionate with myself than I was with others | 0 | 1 | 2 | 3 | 4 |
| 1. I put off taking care of my own health due to time pressure | 0 | 1 | 2 | 3 | 4 |
| 1. Taking care of my needs seemed incompatible with taking care of my staff’s needs | 0 | 1 | 2 | 3 | 4 |

Impact on personal relationships

In the past year my job has:

|  | Not at all true | Somewhat true | Moderately true | Very true | Completely true |
| --- | --- | --- | --- | --- | --- |
| 1. Made it harder for me to develop new meaningful personal relationships | 0 | 1 | 2 | 3 | 4 |
| 1. Made it harder for me to nurture existing personal relationships | 0 | 1 | 2 | 3 | 4 |
| 1. Contributed to conflict in my personal relationship(s) | 0 | 1 | 2 | 3 | 4 |
| 1. Contributed to me feeling more isolated or detached from the people who are most important to me | 0 | 1 | 2 | 3 | 4 |

Demographics

1. What is your age in years?

- 20-29 years old
- 30-39 years old
- 40-49 years old
- 50-59 years old
- 60-64 years old
- 65 years old or older
- Prefer not to answer

1. Please indicate which term best describes your gender identity:

- Woman
- Man
- Non-binary
- Other
- Prefer not to answer

1. Do you identify as a racialized person/person of color?

- Yes
- No
- Prefer not to answer

1. *[Display if answer yes to Q58 - racialized/person of colour]* Do you identify as being a member of any of these population groups? [check all that apply]

- First Nations, Inuk (Inuit) or Métis
- White
- South Asian
- Chinese
- Black
- Filipino
- Arab
- Latin American
- Southeast Asian
- West Asian
- Korean
- Japanese
- Other [enter]
- Prefer not to answer

1. The following questions refer to your working hours before the COVID-19 pandemic and during the peak and non-peak times of the COVID-19 pandemic.
2. Pre-pandemic (i.e., before January 2020), including overtime, how many hours did you usually work per week?

- 0 hours
- 1 to 19 hours
- 20 to 29 hours
- 30 to 39 hours
- 40 to 49 hours
- 50 or more hours
- Prefer not to answer

1. During the peak times of the pandemic (e.g., Omicron wave), including overtime, how many hours did you usually work per week?

- 0 hours
- 1 to 19 hours
- 20 to 29 hours
- 30 to 39 hours
- 40 to 49 hours
- 50 or more hours
- Prefer not to answer

1. During times of lower COVID-19 circulation during the pandemic (e.g., summer 2022), including overtime, how many hours did you usually work per week?

- 0 hours
- 1 to 19 hours
- 20 to 29 hours
- 30 to 39 hours
- 40 to 49 hours
- 50 or more hours
- Prefer not to answer

1. Did you receive remuneration from your employer beyond your usual salary and benefits during the pandemic?

- Yes
- Pay associated with number of hours worked (e.g., overtime pay)
- Pay not associated with number of hours worked (e.g., lump sum for all service provided)
- Time in lieu (e.g., additional vacation)
- Other tangible benefits (e.g., free childcare)
- Other [describe]
- No
- Prefer not to answer

1. What was your total household income in Canadian dollars from all sources before taxes last year?

- Less than $150,000
- $150,000 to $249,999
- $250,000 to $349,000
- $350,000 to $449,000
- More than $450,000
- Prefer not to answer

1. Are you a caregiver for any children less than 18 years of age?

- Yes
- No
- Prefer not to answer

1. Are you a caregiver for any adult dependents?

- Yes
- No
- Prefer not to answer

1. Are there positive things that kept you motivated while working in public health during the pandemic?

- Yes
- No
- Prefer not to answer

*If yes, please describe briefly. (text box – optional question – max 50 characters)*

Survey Submission

1. Do you wish to submit your survey?

- Yes
- No (End survey without submitting responses)

*If participant selects “yes” to submitting the survey, go to Question 53.*

Thank you for participating.

1. Would you like to receive the results of this study?

- Yes → *take to separate survey link to collect name and contact info (email and phone number) (will then be taken to Question 68)*
- No

1. Would you be willing to participate in a key informant interview to better understand your experience of the COVID-19 pandemic and its impacts on your health and well-being?

- Yes → *take to separate survey link to collect name and contact info (email and phone number) (debrief page will be shared after submission of the contact info survey)*
- No *(to debrief page)*
